# A screening tool for attention deficit hyperactivity disorder in children in Saudi Arabia

**DOI:** 10.4103/0256-4947.55321

**Published:** 2009

**Authors:** Ahmad M. Hassan, Fatima Al-Haidar, Fateh Al-Alim, Othman Al-Hag

**Affiliations:** a Department of Neurosciences, King Faisal Specialist Hospital and Research Centre, Riyadh, Saudi Arabia; b Department of Psychiatry, Riyadh Military Hospital, Riyadh, Saudi Arabia; c Department of Psychiatry, King Khalid University Hospital, King Saud University, Riyadh, Saudi Arabia; d Riyadh Mental Hospital, Riyadh, Saudi Arabia

## Abstract

**BACKGROUND AND OBJECTIVES:**

A clinically validated attention deficit hyperactivity disorder (ADHD) scale in Arabic for evaluating children in Saudi Arabia who might be suspected of having ADHD is lacking. Thus, we studied the validity of an Arabic version of the ADHD Rating Scale in discriminating children with an ADHD diagnosis from normal children or from those with non-ADHD psychiatric diagnoses, including mental retardation.

**METHODS:**

The guardians of 119 children provided demographic data and completed the standardized Arabic version of the ADHD Rating Scale on their children, who were either normal, had a diagnosis of ADHD, or had a non-ADHD psychiatric diagnosis. The mean rating scores of the groups were compared, and the cutoff points were calculated for both sexes.

**RESULTS:**

The scores discriminated children with ADHD diagnosis (mean and [SD], 28 [6.288]) from normal children (10.93 [8.009]), and those with a non-ADHD psychiatric diagnosis (16.63 [8.865]). ADHD cutoff points were obtained for male (23.5) and female (22.5) children. Psychosocial characteristics associated with children having ADHD were not associated with the diagnosis of ADHD.

**CONCLUSION:**

The ADHD Rating Scale (Arabic version), in terms of either the grand total score or the total score of each of its two subscales, demonstrated concurrent and discriminant validity by discriminating children with ADHD from other clinical and non-clinical children groups. The study obtained cutoff points for both sexes based only on the grand total score of the scale because of the relatively small sample size. Replication of the study, utilizing a larger sample and eliciting ratings from both parents and teachers, is recommended.

There is a general consensus that well standardized behavior rating scales are essential in the evaluation of children with attention deficit hyperactivity disorder (ADHD). However, such rating scales have their limitations. They are subject to biases in opinion, such that various characteristics of the informants, including their education and emotional state at the time of conducting the ratings, may influence the ratings. Also, rating scales may fail to assess factors influencing a child's behavior.^1^ Other sources of limitation are inherent to the construction of the rating scales themselves, including the specificity of wording and the breadth of response scaling. In addition, demographic variables may produce situational variation in a child's behavior^1^ or in the way children's parents, for example, may perceive and rate the behavior of their children.^2^ There are several types of behavior rating scales that have been used in assessing ADHD symptoms in children and adolescents. However, there is no well-validated scale in Arabic for children. Our study aimed at validating children in Saudi Arabia one of the most commonly used scales, the ADHD Rating Scale^3^ for use in screening children with ADHD symptoms. The scale was selected because of its established ability to discriminate ADHD children from those in clinical and normal populations,^1^ and because it provides a direct rating of the essential symptoms of the disorder as per the diagnostic criteria of the Diagnostic and Statistical Manual (version 3, revised (DSM-III-R).^4^ This study predominantly focused on examining the validity of the grand total score of the ADHD Rating Scale (Arabic Version) in discriminating children with ADHD from either normal children or children with any of a set of psychiatric diagnoses other than ADHD.

## METHODS

The ADHD Rating Scale (Arabic version) (Figure 1) is the translated and linguistically standardized version of the original ADHD Rating Scale.^3^ It contains 14 items, each of which is a 4-point scale (0-3) arranged in the following format: not at all, just a little, pretty much, very much. The scale, in its original language, has an established test-retest reliability and internal consistency reliability. In addition, it has established construct, discriminant, and concurrent validity measures. The completion time for the scale is 5 minutes. Finally, the administration of the scale can yield three scores: a) grand total score, b) inattention-restlessness sub-scale score (containing items that are intended to screen specifically the symptoms that are assumed to be pertinent to the factor of inattention/restlessness), and c) impulsivity-hyperactivity sub-scale score (containing items that are intended to screen specifically the symptoms that are assumed to be pertinent to the factor of impulsivity/hyperactivity).

**Figure 1:**
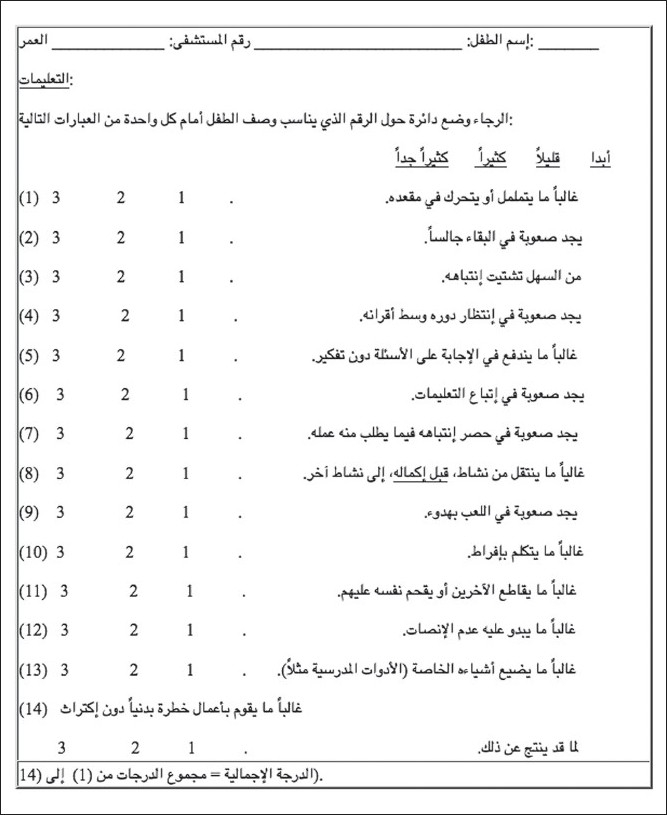
Attention deficit hyperactivity disorder rating scale (Arabic version).

The Department of Translation, King Saud University was consulted for the processes of translation and back-translation, while the technical adaptation of the scale was performed by the researchers who, after a pilot study (n=56), examined and adapted the Arabic version to ensure that it preserved the format, consistency and content of the English version of the scale. Personal and demographic data about the participants in the study, including information about the child, his or her parents, siblings, and the principal guardian if not one of the parents were collected. These include family structure and size, educational level of the child and his parent/guardian, status of parental marriage (i.e., living together or separated), the other socioeconomic data.

The ADHD Rating Scale^3^ is based on the DSM-III-R construct of attention deficit hyperactivity disorder, since it was the current DSM edition at the time of publishing the rating scale. The researchers in this study found it appropriate to use the scale at the onset of the study in early 1995 by which time the 4th edition was published,^5^ since there were no significant differences, as far as the ADHD construct was concerned, between the two editions. For example, the DSM-IV criteria require impairment across settings, while this study focused on both parent ratings and clinical assessment since access to the school setting was not attainable. However, while the DSM-III-R did not identify any subtypes of ADHD, the DSM-IV has conceptualized ADHD as consisting of three subtypes: Inattentive, Hyperactivity-Impulsivity, and Combined.^6^

In 1995, the original ADHD Rating Scale^3^ was translated into Arabic and was pilot studied (n=56) by three psychiatrists and a clinical psychologist from three major mental health centers in Riyadh, Saudi Arabia (The Departments of Psychiatry in Riyadh Military Hospital and King Khalid University Hospital and Riyadh Mental Health Hospital). The main study subjects were freshly recruited on a first-come first-included basis at the child mental health clinics of the three participating mental health centers. The guardians of children participating in the study were interviewed to elicit their consent for participation in the study, and they completed the structured, purpose-built data collection form. The guardians of the children were then asked to rate their children using the standardized Arabic version of the ADHD Rating Scale. In two of the three centers, the clinician who confirmed the psychiatric diagnosis of the children was always different from the clinician who processed the completion of the data collection form with the guardian of the respective child and who handed the ADHD Rating Scale (Arabic version) to the respective child guardian to complete. The sub-sample of normal children was recruited from among the child population visiting the immunization clinic in one of the three mental health centers participating in the study. The recruited children were subsequently screened by one of the psychiatrists.

This study sample included 119 children, of which 90 (89.1%) had various psychiatric diagnoses confirmed by a psychiatrist, and 29 (10.9%) were clinically screened by a psychiatrist to be normal children. The normal children as well as those with psychiatric diagnoses were subsequently designated to the following diagnostic groups: normal, ADHD diagnosis only (ADHD Dx), ADHD diagnosis combined with another psychiatric (Axis I) diagnosis (ADHD+Psy Dx), Psychiatric (Axis I) diagnosis only (Psy Dx) and mental retardation only (MR).

The data were tabulated and analyzed using the Statistical Package for Social Sciences, version 10 (SPSS 10). The chi-square test was used for categorical variables, the t test for numerical variables, and one-way analyses of variance (ANOVA) and Scheffé test for more than two-group analysis. For determining the cutoff points, including sensitivity and specificity measures, based on the grand total score of the scale, the receiver operating characteristics (ROC) curve was plotted.

## RESULTS

The study included 119 children (80 males, 39 females) (Table 1) with a mean age of 8 years (range, 3 to 13 years). Of these 119 children, 112 (94.2%) were 5 years of age or older. Of the whole sample, 58 (48.7%) had no schooling, 55 (46.1%) were in primary education, and 4 (3.4%) had no information on their education. There were no significant differences between the groups in most demographic variables, but the diagnostic groups differed significantly (*P* =.0001) in terms of the children's years of formal schooling, with the children in the normal group having a higher mean school grade than that of the ADHD plus and the mental retardation groups.

**Table 1:** Sex distribution of the diagnostic groups.

Diagnostic group	Males (%)	Females (%)	Total (%)
Normal	15 (12.6)	14 (11.8)	29 (24.4)
ADHD diagnosis only	13 (10.9)	05 (04.2)	18 (15.1)
ADHD+ psychiatric diagnosis	11 (09.2)	02 (01.7)	13 (10.9)
Psychiatric diagnosis	34 (28.6)	12 (10.1)	46 (38.7)
Mental retardation	07 (05.9)	06 (05.0)	13 (10.9)

Total	80 (67.2)	39 (32.8)	119 (100)

Children in the ADHD diagnosis only and the ADHD plus psychiatric diagnosis groups had significantly higher grand total scores compared to children in the normal group (*P* <.001) or in either the mental retardation (*P* <.05) or the psychiatric diagnosis (*P* <.001) groups (Table 2). The mean (SD) scores for each diagnostic group were as follows: AHDH diagnosis 28.0 (6.288), normal 10.93 (8.009), psychiatric diagnosis 16.63 (8.865), mental retardation 17.15 (9.317). The receiver operating characteristics curve showed that the grand score had a sensitivity of 0.742 and a specificity of 0.773, while the overall cutoff point for both sexes was 23.5 (23.5 for males and 22.5 for females) (Figure 1). The Scheffé test results showed that the “inattention-restlessness” mean scores of children in the ADHD diagnosis only and the ADHD plus psychiatric diagnosis groups differed significantly (*P* <.001) from that of the children in either the normal (*P* <.001), the psychiatric diagnosis (*P* <.001), or the mental retardation (*P* <.01) groups (Table 3). The mean score of the ‘impulsivity-hyperactivity’ subscale differentiated between the children with ADHD and children in other groups of the study (*P* <.001) (Table 4). However, the ‘impulsivity-hyperactivity’ mean scores of the ADHD diagnosis only and the ADHD plus psychiatric diagnosis groups were significantly larger than that of either the normal (*P* <.001), the psychiatric diagnosis (*P* <.01), or the mental retardation (*P* <.05) groups.

**Figure 1:**
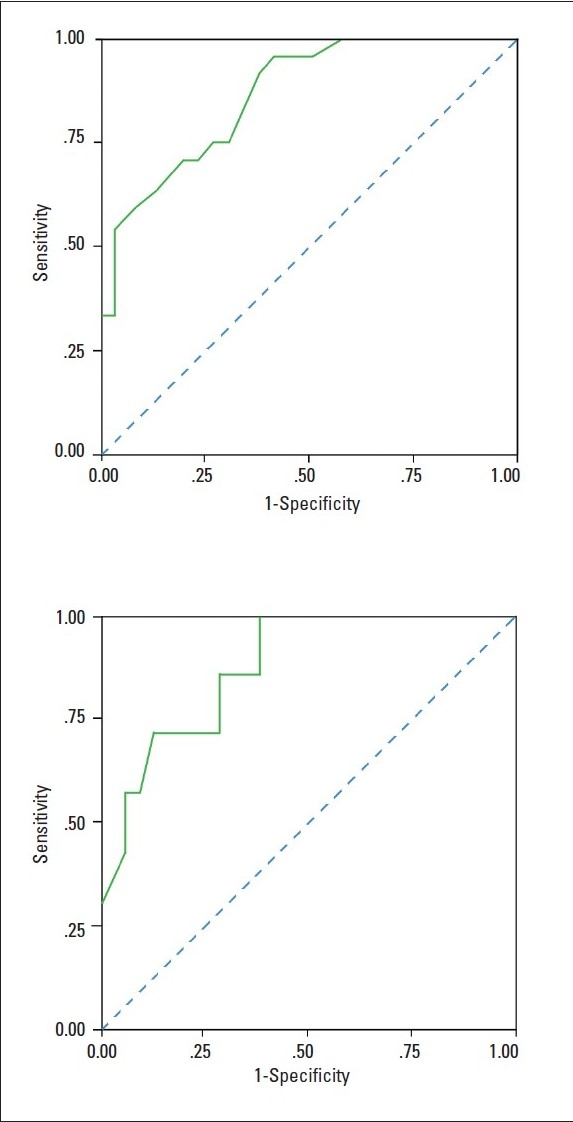
Receiver operating characteristics curves for males (top) and females (bottom). Values along the 45-degree diagonal indicate results no better than chance for detecting disease.

**Table 2:** Multiple comparisons of diagnostic group mean grand total scores on the ADHD Rating scale (Arabic version).

	**Normal**			
				
ADHD diagnosis only	a	**ADHD diagnosis only**		
				
ADHD+ psychiatric diagnosis	a	0.998	**ADHD+other psychiatric diagnosis**		
				
Psychiatric diagnosis	0.076	a	a	**Psychiatric diagnosis**
				
Mental retardation	0.270	b	b	1.000

^a^*P*<.001

^b^*P*<.05 (Scheffé Test)

**Table 3:** Multiple comparisons of diagnostic group mean scores on the “Inattention- Restlessness” sub-scale of the ADHD Rating Scale (Arabic Version).

	**Normal**			
					
ADHD diagnosis only	a	**ADHD diagnosis only**		
					
ADHD+ psychiatric diagnosis	a	0.991	**ADHD+other psychiatric diagnosis**	
				
Psychiatric diagnosis	0.610	a	a	**Psychiatric diagnosis**
				
Mental retardation	0.910	b	b	1.000

^a^*P*<.001

^b^*P*<.01 (Scheffé Test)

**Table 4:** Multiple comparisons of diagnostic group mean scores on the “Impulsivity- Hyperactivity” sub-scale of the ADHD Rating Scale (Arabic Version).

	**Normal**			
				
ADHD diagnosis only	a	**ADHD diagnosis only**		
				
ADHD+ psychiatric diagnosis	a	1.000	**ADHD+other psychiatric diagnosis**	
				
Psychiatric diagnosis	b	b	b	**Psychiatric diagnosis**
				
Mental retardation	c	c	c	0.999

^a^*P*<.001

^b^*P*<.01

^c^*P*<.05 (Scheffé Test)

## DISCUSSION

A valid and reliable behavioral rating scale for screening of children suspected of having ADHD is an essential step toward accurate diagnosis and optimal management. The need for such a scale in Saudi Arabia can hardly be over-emphasized. Our results show that the grand total scores obtained by the children on the ADHD Rating Scale (Arabic version) differentiated between the children with ADHD, whether as a pure clinical condition or as a co-morbidity, and the children who were either normal or had a psychiatric diagnosis other than ADHD, including the mental retardation diagnosis.

Two prevalence studies on ADHD in Saudi Arabia reported rates of 12% and 16.5%.^7^ Regardless of the accuracy of such a high rate of prevalence for this disorder, observations and discussions with various mental health workers and school teachers indicate that manifestations of at least ADHD-like symptoms are common and frequent features among a notable proportion of the childhood population in primary school settings. On the other hand, there is as yet no rating scale for screening ADHD symptoms that has clinically proven validity measurement indices that can validly and reliably discriminate children with ADHD symptoms from normal children and children with such conditions as anxiety, depression, mental retardation, or other childhood mental disorders. The few ADHD rating scales existing in Saudi Arabia are merely translations of foreign rating scales. They have not been sufficiently studied in terms of their validity and reliability measures. For example, none of the local rating scales has a proven validity based on a comparison with a gold standard, such as a valid clinical diagnosis or a similar rating scale with empirically proven validity measures.

The original scale was developed and standardized for obtaining a direct rating of the essential symptoms of the disorder from both parents and teachers.^3^ However, ratings were obtained from parents only in this study, a situation that was largely due to logistical constraints such that access to school settings at the time of the study was complicated by official and administrative obstacles. A future replication of the study is recommended whereby ratings are to be obtained from both parents and teachers.

The results of this study indicate that ADHD is not restricted to a particular social strata, family type, family size, or to parents with a certain educational level. For example, it was found that parents who rated the symptoms of their children on the ADHD Rating Scale (Arabic version) above the cutoff for ADHD diagnosis were of various educational levels, ranging from illiteracy to post-graduate education. This finding also indicates that parents' recognition of the behavioral manifestations of the disorder is irrelevant to their educational level.

Not surprisingly, the study groups differed significantly in terms of the school grade that a child had achieved (i.e., years of schooling). Children who were mentally retarded and children with ADHD combined with another Axis I psychiatric diagnosis (i.e., co-morbidity) had a significantly lower grade level. An examination of the data has shown that the mentally retarded children either did not attend any school, or they attended what is locally termed “special schools” where the mentally retarded children attend ‘school time’ during daytime with very little to do except playing games, and they usually do not progress from one grade to a higher one.

Although the parents' educational level did not show a significant group difference in this study, a trend was observed for some parents with high educational level to rate their children relatively higher on ADHD symptoms, compared to those with low or no education.^7^ This observation is thought relevant to the theoretical assumption that highly educated parents are more likely to belong to middle or high socioeconomic strata and are likely to be more observant and probably more critical of their children's behavioral disturbances, with a lower tolerance for such behaviors, compared to parents in a low socioeconomic stratum and with low or no education. For example, Weckerly, et al^2^ found that caregiver years of education was significantly positively associated with the “inattention” but not with the “hyperactivity-compulsivity” symptoms of ADHD in a sample of high-risk youths in public service sectors. However, the sample size of this study is deemed relatively small, such that a larger sample size is recommended in future studies, which may allow for more adequate exploration of the effect of parents' educational level on the way children were rated on the scale.

In this study, the psychiatrist who reached the diagnosis for a child is different from the one who supervised the completion of the data collection form by the parent of the same child. Some of the guardians who rated their children on the ADHD Rating Scale (Arabic version) preferred to complete the scale at home due to time constraints. This arrangement did not seem to have influenced the quality of their rating in any inappropriate way. However, a future similar study would be expected to apply additional controls on the study, specifically by blinding the process of diagnosing children from the process of rating the same children, preferably with inter-rater protocols.

The sample of this study is largely a clinical sample. Future studies on the prevalence of ADHD in the Saudi community, involving children recruited from their community settings, are highly recommended. However, unless a solid screening tool with strong validity indices is established, community screening for prevalence of ADHD may be misleading. Therefore, it is recommended that a future similar study administer the validated ADHD rating scale to a larger number of participants. Such an exercise may re-confirm or modify the cutoff points established in this study.

In summary, the ADHD Rating Scale (Arabic version) successfully differentiated children with a clinically proven ADHD diagnosis from both normal children and children with non-ADHD psychiatric diagnoses that include mental retardation. The “inattention” and “impulsivity-hyperactivity” subscales were equally successful in such a differentiation. The study re-emphasized the importance of developing and validating ADHD rating scales against some solid gold standards before administering them for obtaining prevalence rates of the disorder in communal settings. The acknowledged limitations, such as small sample size and utilization of a rating scale that is probably out-dated, may not overshadow the fact that the study probably provided the clinical and investigative grounds for an improved research work on ADHD in Saudi Arabia in the recent future.

## References

[1] Barkley RA (1990). Attention Deficit Hyperactivity Disorder: A Handbook for Diagnosis and Treatment.

[2] Weckerly J, Aarons GA, Leslie LK, Garland AF, Landsverk J (2005). Attention on inattention: the differential effect of caregiver education on endorsement of ADHD symptoms. Dev Behav Pediatr.

[3] DuPaul GJ, Russell A Barkley (1990). The ADHD Rating Scale: normative data, reliability, and validity. Unpublished manuscript, University of Massachusetts Medical Center, Worcester.

[4] American Psychiatric Association (1987). Diagnostic and statistical manual of mental disorders.

[5] American Psychiatric Association (1994). Diagnostic and statistical manual of mental disorders.

[6] Collett BR, Ohan JL, Myers KM (2003). Ten-year review of rating scales V: scales assessing attention-deficit hyperactivity disorder. J Am Child Adolesc Psychiatry.

[7] AlHamed JH (2001). Attention-deficit hyperactivity disorder among male primary school children: prevalence and some associated psycho-social factors [dissertation].

[8] DuPaul GJ, Power TJ, Anastopoulos AD, Reid R ADHD Rating Scale-IV: Checklist, Norms, and Clinical Interpretation.

